# Assessing the Functional Role of Leptin in Energy Homeostasis and the Stress Response in Vertebrates

**DOI:** 10.3389/fendo.2017.00063

**Published:** 2017-04-07

**Authors:** Courtney A. Deck, Jamie L. Honeycutt, Eugene Cheung, Hannah M. Reynolds, Russell J. Borski

**Affiliations:** ^1^Department of Biological Sciences, North Carolina State University, Raleigh, NC, USA

**Keywords:** leptin, energy homeostasis, stress, teleosts, metabolism, cortisol, appetite

## Abstract

Leptin is a pleiotropic hormone that plays a critical role in regulating appetite, energy metabolism, growth, stress, and immune function across vertebrate groups. In mammals, it has been classically described as an adipostat, relaying information regarding energy status to the brain. While retaining poor sequence conservation with mammalian leptins, teleostean leptins elicit a number of similar regulatory properties, although current evidence suggests that it does not function as an adipostat in this group of vertebrates. Teleostean leptin also exhibits functionally divergent properties, however, possibly playing a role in glucoregulation similar to what is observed in lizards. Further, leptin has been recently implicated as a mediator of immune function and the endocrine stress response in teleosts. Here, we provide a review of leptin physiology in vertebrates, with a particular focus on its actions and regulatory properties in the context of stress and the regulation of energy homeostasis.

## Introduction

Leptin is a class I helical cytokine encoded by the *obese* gene (*ob*) that has typically been characterized as an adipostat, circulating in proportion to the quantity of white adipose tissue and relaying information regarding the energy status of the animal to the central nervous system (1, 2). In mammals, leptin is pleiotropic, regulating a multitude of physiological processes including appetite, lipid metabolism, growth, reproduction, stress, and immune function [reviewed in Ref. (3)]. The function of leptin has been less extensively studied in non-mammalian vertebrates; however, there is growing evidence in teleosts that leptin may play a greater role as a glucoregulatory hormone than an adipostat in this group of vertebrates. Studies on the interactions between leptin and the stress axis as well as the immune system, however, suggest that some of the actions of leptin may be conserved between fish and mammals despite the low sequence conservation between these two groups. Here, we provide an overview of what is known about the role of leptin in regulating energy homeostasis and the stress response in teleost fishes and compare this to the known effects of leptin in mammals and other vertebrate groups.

## Leptin Characterization, Distribution, and Signaling

### Orthology in Vertebrates

**Figure 1:**
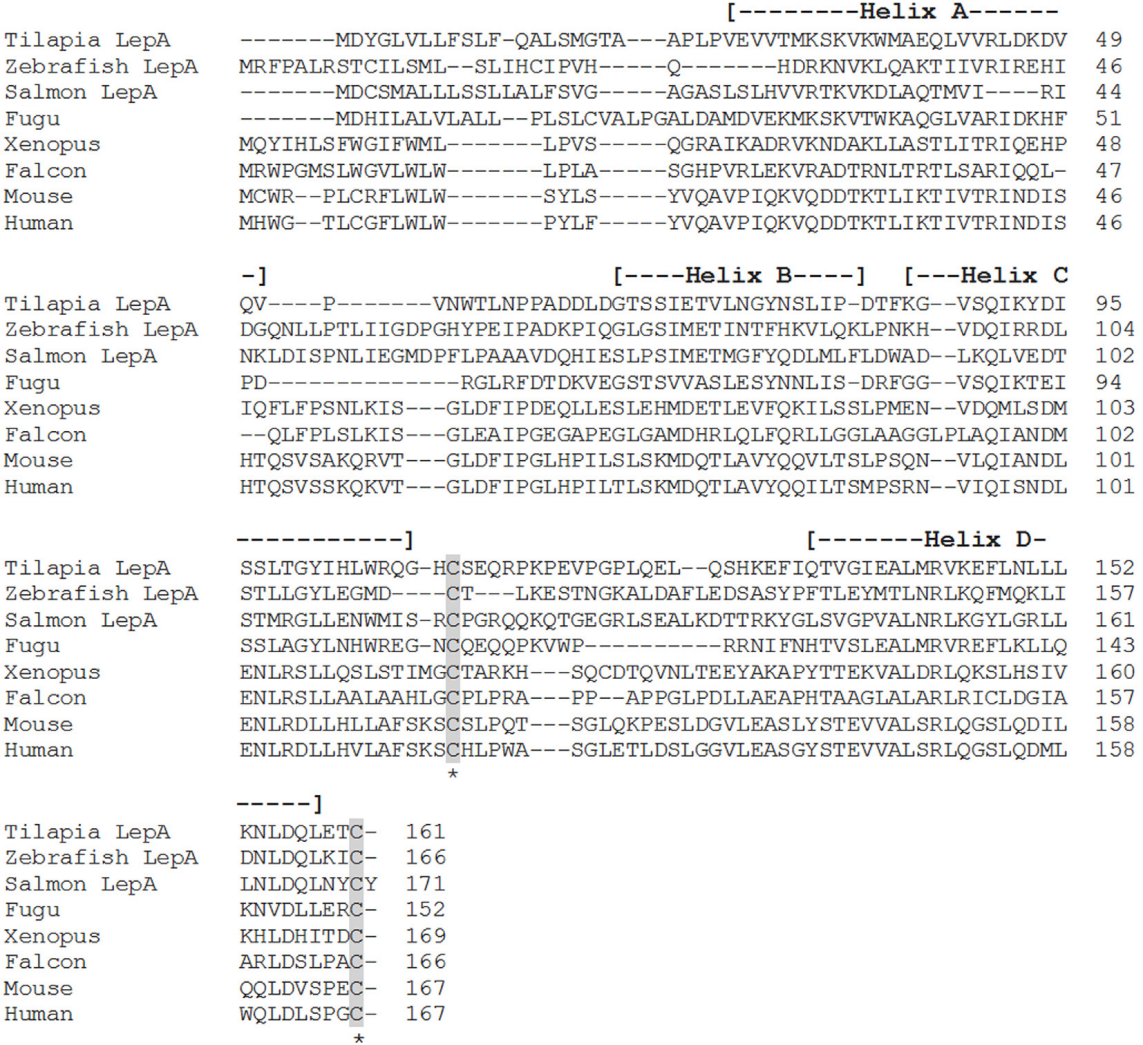
**Alignment of teleost leptin A (LepA) with the leptin homologs from other vertebrate classes**. Accession numbers: tilapia LepA, AHL37667.1; zebrafish LepA, NP_001025357.2; salmon LepA, ACZ02412.1; fugu, NP_001027897.1; *Xenopus*, NP_001089183.1; falcon, NP_001298279.1; mouse, NP_032519.1; human, NP_000221.1. Shaded areas represent the conserved cysteine residues required for the formation of the disulfide bridge. The four alpha-helices are indicated by dashed lines within the parentheses.

Leptin was first cloned in the mouse by Zhang et al. (1) and has since been identified in all extant vertebrate groups examined to date. Following the discovery of leptin in the mouse, orthologs were identified in several other mammalian species (4); however, attempts to isolate a putative leptin sequence in non-mammalian vertebrates were largely unsuccessful. It was not until 2005, over a decade after its discovery in mammals, that a leptin homolog was cloned in a non-mammalian species, the Japanese pufferfish [*Takifugu rubripes* (5)]. This delay was due to the low amino acid identity (often less than 30%) between vertebrate leptin sequences (6) (Figure 1). The deduced primary structure of the pufferfish leptin (pLep) shared only 13.2% identity with human leptin; however, three-dimensional modeling suggested a strong conservation of tertiary structure with mammalian leptins, as pLep also possesses four α-helices (5). Further, the amino acid sequence of pLep contained two cysteine residues to form the disulfide bridge between α-helices C and D, a highly conserved element of vertebrate leptins (5).

**Figure 2:**
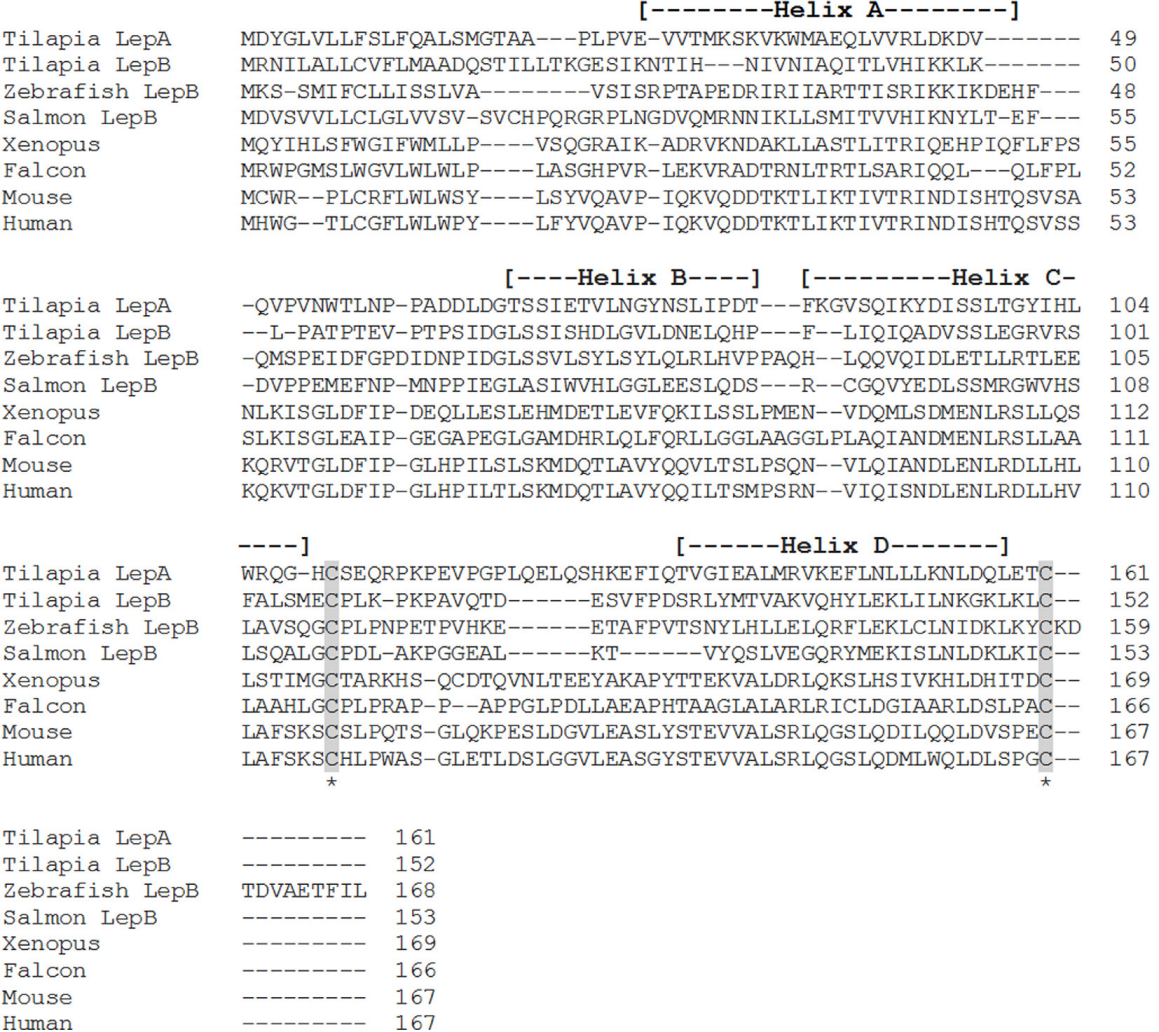
**Alignment of teleost leptin B (LepB) with the leptin homologs from other vertebrate classes, tilapia leptin A (LepA), has been included for comparison**. Accession numbers: tilapia LepA, AHL37667.1; tilapia LepB, AHL37668.1; salmon LepB, NP_001266063.1; zebrafish LepB, NP_001025357.2; *Xenopus*, NP_001089183.1; falcon, NP_001298279.1; mouse, NP_032519.1; human, NP_000221.1. Shaded areas represent the conserved cysteine residues required for the formation of the disulfide bridge. The four alpha-helices are indicated by dashed lines within the parentheses.

Shortly after the identification of pLep, a leptin homolog was cloned in an amphibian, *Xenopus laevis*, that shared 35 and 13% amino acid identity with human and pLeps, respectively (7) (Figure 1). Putative leptin sequences have also been identified in the tiger salamander [*Ambystoma tigrinum* (8)] and in the green Anole lizard [*Anolis carolinensis* (9)], both of which show low amino acid identity to human leptin. In teleosts, leptin orthologs have now been characterized in striped bass [*Morone saxatilis* (10)], common carp [*Cyprinus carpio* (11)], rainbow trout [*Oncorhynchus mykiss* (12)], zebrafish [*Danio rerio* (13)], Atlantic salmon [*Salmo salar* (14)], orange-spotted grouper [*Epinephelus coioides* (15)], Japanese medaka [*Oryzias latipes* (13, 16)], yellow catfish [*Pelteobagrus fulvidraco* (17)], Nile tilapia [*Oreochromis niloticus* (18)], Jian carp [*C. carpio* var. Jian (19)], Arctic charr [*Salvelinus alpinus* (20)], grass carp [*Ctenopharyngodon idella* (21)], silver carp [*Hypophthalmichthys molitrix* (21)], chub mackerel [*Scomber japonicus* (22)], mandarin fish [*Siniperca chuatsi* (23)], and white-clouds mountain minnow [*Tanichthys albonubes* (24)]. These teleost leptins all have low sequence conservation with mammals, varying from 13 to 25% amino acid identity (Figures 1 and 2); however, each one is composed of two exons separated by a short intron, contains the cysteine residues required for formation of the disulfide bridge, and is predicted to have retained the four-helix tertiary structure characteristic of mammalian leptins. Even within the teleost lineage, there is often low amino acid identity between species (<50%), unless the species are closely related, such as within the salmonids or cyprinids.

### Paralogs in Teleosts

In certain teleost species, two leptin paralogs have been identified. This is a common feature of teleostean class I cytokines, resulting from the genome duplication that occurred in the teleost lineage (6, 25). Zebrafish (*D. rerio*), Japanese medaka (*O. latipes*), orange-spotted grouper (*E. coioides*), Nile tilapia (*O. niloticus*), Mozambique tilapia (*Oreochromis mossambicus*), chub mackerel (*S. japonicus*), mandarin fish (*S. chuatsi*), and white-clouds mountain minnow (*T. albonubes*) have all been shown to possess two separate leptin proteins, leptin A (LepA) and leptin B (LepB) (13, 15, 18, 19, 22–24, 26) (Figures 1 and 2). The amino acid identity between LepA and LepB within each species is low, ranging from 18 to 30%, and phylogenetic analysis shows that the two genes form separate branches (18, 19, 24, 27). Due to the additional genome duplications that occurred within the salmonid and cyprinid lineages, a number of species including the common carp (*C. carpio*), Atlantic salmon (*S. salar*), goldfish (*Carassius auratus*), rainbow trout (*O. mykiss*), and Jian carp (*C. carpio* var Jian) possess up to four leptin paralogs, two LepA genes, and either one or two LepB genes (11, 14, 19, 28, 29). The two LepA sequences and the two LepB sequences in these species share higher amino acid identities than is seen between the A and B forms (ranging from 71 to 83%); thus, the nomenclature typically used is leptin A1 and A2 and leptin B1 and B2 (13, 14, 28, 29).

### Tissue Distribution in Teleosts

Unlike in mammals where leptin is produced predominantly in adipose tissue, teleost leptins often have the highest mRNA expression levels in liver, with most species having low or non-existent leptin expression in adipose tissue. Other sites of expression in teleosts are the brain, gonads, muscle, and kidney; however, this can vary widely between species (10–24, 26). In some instances, the tissue distribution between paralogs within a single species differs, and it has been suggested that *lepa* is more prominent in the liver, while *lepb* is predominantly expressed in the gonads, thus indicating divergent roles of the two paralogs (13, 29). However, studies on LepB are limited, and this differential expression pattern is not consistent across species, with most showing substantial overlap in the tissue expression patterns for the two forms. Regardless, *lepa* appears to be the predominantly expressed form in most species examined (15, 16, 24) showing 10–100 times greater tissue mRNA copy number than *lepb* and hence likely reflecting the major source of circulating leptin (26).

### Receptor and Signaling Pathways

The leptin receptor (LepR) is part of the glycoprotein 130 family of cytokine receptors, which utilize gp130 as a signal transducer to activate signaling pathways within the cell, typically the Janus kinase/signal transducers and activators of transcription (JAK/STAT) pathway (30, 31). Signaling *via* this pathway has been observed in the pituitary of both mammals and frogs, suggesting conservation of this signaling mechanism for leptin across vertebrate groups (30–32). Although sharing low identity with mammalian receptors (<30%), teleost LepRs show genomic synteny with the human receptor and possess the functionally important JAK- and STAT-binding domains that are largely conserved within vertebrates (14–18, 33–35). In teleosts, *lepr* mRNA is ubiquitously expressed, with higher levels typically being observed in the pituitary, hypothalamus, and gonads, suggesting that these are prominent sites of leptin action (14–18, 28, 33–35). Indeed, leptin regulates glucose sensing in the hypothalamus and hindbrain of rainbow trout (*O. mykiss*) both *in vitro* and *in vivo* (36, 37). These actions were attenuated when leptin was administered in combination with either a phosphoinositide-3-kinase or JAK2 inhibitor, indicating involvement of these pathways in leptin signaling (36, 37). Further evidence for leptin signaling *via* the JAK/STAT pathway comes from the increase in Akt and STAT3 phosphorylation observed in trout hypothalamic cells following incubation with leptin (38). The lipid regulatory activity of heterologous leptin on hepatocytes and ovarian follicular cells of yellow catfish (*P. fulvidraco*) is attenuated by JAK/STAT inhibitors, reiterating a role for this pathway in leptin signaling (39). Leptin has also been shown to act on the pituitary of tilapia (*O. mossambicus*) to stimulate prolactin (PRL) release through activation of the extracellular signal-related kinase (ERK) pathway (40) and on the liver of the hybrid striped bass [*Morone chrysops x Morone saxatilis* (41)] and Mozambique tilapia [*O. mossambicus* (42)] to regulate growth hormone (GH) receptors and insulin-like growth factors (IGFs), although the signaling pathways have yet to be determined. Albeit studies assessing the function of leptin in teleosts are limited, existing data suggest that the sites of leptin action and the signaling pathways responsible for eliciting its effects may be conserved with that of other vertebrate systems. Further investigations are required to elucidate the full complement of intracellular pathways mediating leptin action(s).

## Leptin Energy Homeostatic Actions

### Feeding

Leptin is renowned for its role in regulating food intake and body mass (43). Secreted primarily from adipose tissue in mammals, leptin serves as a lipostatic signal and conveys critical information regarding metabolic state to the brain (44, 45). As lipid stores accumulate and circulating leptin rises, the hormone enhances energy expenditure and reduces food intake by stimulating anorexic proopiomelanocortin/cocaine and amphetamine-related transcript neurons and inhibiting orexigenic neuropeptide Y/agouti-related protein neurons (46–50). Leptin-deficient pathologies are typically accompanied by hyperphagia and obesity [reviewed in Ref. (45, 49, 51)]. The anorexigenic properties of leptin have been well characterized in the context of leptin deficiency through experimental administration to obese, leptin-deficient *ob*/*ob* mice, as well as leptin-deficient humans, resulting in the reduction of food intake and body mass (52, 53).

In some fishes, leptin demonstrates a marked postprandial elevation [(54, 55); reviewed in Ref. (56, 57)] in accordance with the mammalian paradigm. Further, the administration of leptin *via* injection has been shown to reduce food intake in goldfish [*C. auratus* (58, 59)], rainbow trout [*O. mykiss* (12, 36)], grass carp [*C. Idella* (21)], Atlantic salmon [*S. salar* (60)], and striped bass [*M. saxatilis* (10)]. Properties similar to that of leptin-related pathologies initially observed in the *db*/*db* mouse have also been reported in a LepR-deficient medaka [*O. latipes* (61)]. This mutant line showed consistently elevated hypothalamic activity of orexigenic neuropeptides, suppression of anorexigenic neuropeptides, and increased food intake, suggesting a similar regulatory role for leptin in appetite suppression in fishes. While the anorexigenic properties of leptin would also suggest potentially concurrent lipostatic properties as seen in mammals, no changes in adiposity were observed in leptin receptor-deficient strains of zebrafish (62), and other species exhibit inconsistent correlations between fat deposition and leptin expression, e.g., during fasting leptin rises in fish as adiposity declines, while it declines with fasting and lipid stores in mammals (38, 42, 63–65). Nonetheless, the anorexigenic properties of leptin appear well conserved among vertebrates.

### Metabolism

Leptin regulates energy availability in mammals by mobilizing lipid stores (66) and stimulating the oxidation of fatty acids (67). It also induces hypoglycemia by enhancing glucose uptake into peripheral tissues (68) and elevates metabolic rate in muscle and liver (69). Studies on the metabolic actions of leptin in other vertebrate classes are limited (Table 1) leading to difficulties in elucidating whether leptin evolved primarily as a lipolytic agent or if its basal metabolic functions are more glucoregulatory in nature. Teleosts appeared relatively early in the vertebrate lineage, and thus, understanding the role of leptin in regulating metabolic pathways in these fish could provide valuable insights into the evolution of energy homeostasis in vertebrates. The existing data in teleosts are equivocal, with lipolytic actions being reported in response to leptin treatment in some species, while in others, leptin instead stimulates glycogen depletion and increases plasma glucose (Table 2).

**Table 1:** **Comparison of the source of leptin, response to fasting, and effects on appetite, energy metabolism, glycemia, and metabolic rate in the different vertebrate classes based on current knowledge**.

Leptin effects	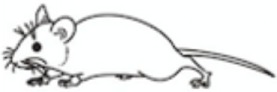	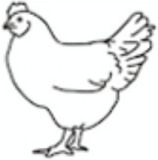	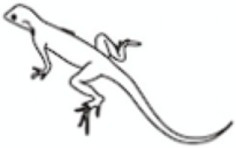	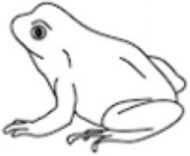	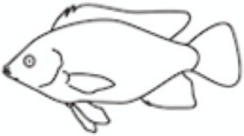
Source	Adipocytes	Adipocytes and hepatocytes	Hepatocytes	?	Hepatocytes
Appetite	Anorexigenic	Anorexigenic	Anorexigenic	Anorexigenic	Anorexigenic
Fasting	Levels decline	?	?	?	Levels elevate^a^
Metabolic rate	Elevates	?	?	Elevates	?
Energy mobilization	Lipolytic	?	Glycogenolytic	?	Glycogenolytic
					Lipolytic^b^
Glycemia	Hypoglycemic	?	Hyperglycemic	?	Hyperglycemic
					Hypoglycemic^b^

*?, unknown*.

*^a^Predominant response but does not occur in all species*.

*^b^Response varies between species*.

**Table 2:** **Different effects of leptin on appetite, energy metabolism, and glycemia as well as the response to fasting in various teleost species**.

Species^a^	Leptin source	Appetite	Energy metabolism	Glycemia	Fasting	Reference
Grass carp (*Ctenopharyngodon idella*)	Carp	Anorexigenic	↑ lipolytic enzyme mRNA	?	?	Li et al. (21)
Catfish (*Pelteobagrus fulvidraco*)	Human	?	↑ lipolytic enzyme mRNA	?	?	Song et al. (39); Zhang et al. (70)
			↑ enzyme activity			
			↓ triglycerides			
Striped bass (*Morone chrysops x Morone saxatilis*)	Human	Anorexigenic	?	?	↓ *lep* mRNA	Won et al. (10)
Tilapia (*Oreochromis mossambicus*)	Tilapia	?	↓ glycogen	Hyperglycemic	↑ *lepa* mRNA	Baltzegar et al. (71); Douros et al. (42)
			↓ lipolytic enzyme mRNA		↑ plasma leptin A	
Rainbow trout (*Oncorhynchus mykiss*)	Salmonid	Anorexigenic	↓ glycogen	Hyperglycemic	↑ plasma Lep	Murashita et al. (12); Kling et al. (72); Aguilar et al. (36, 37)
Goldfish (*Carassius auratus*)	Human	Anorexigenic	↑ glycogen	Hypoglycemic	No effect?	de Pedro et al. (58); Vivas et al. (59); Tinoco et al. (28)
			↓ lipids			

*The source of leptin indicates whether homologous or heterologous leptin was used in the study*.

*^a^Species were chosen to highlight the disparate effects of leptin observed in teleosts*.

Leptin actions appear to agree with the classic mammalian paradigm in grass carp (*C. Idella*), wherein it induces a decrease in the hepatic stearoyl-coA desaturase-1 mRNA, an enzyme involved in the synthesis of fatty acids, while simultaneously increasing the mRNA level of hormone-sensitive lipase (*hsl)* (21). Fatty acid levels were not measured in these studies; however, an overall effect on lipid regulation cannot be ascertained. Nonetheless, human leptin increases activity and mRNA levels of lipolytic enzymes in catfish (*P. fulvidraco*) hepatocytes and ovarian follicular cells, which paralleled a decrease in overall lipid content, suggesting a lipolytic action of the hormone (39, 70). Further, human leptin increased the mRNA levels of various lipolytic genes, decreased the levels of lipogenic genes, and decreased overall triglyceride content in hepatocytes of the goby [*Synechogobius hasta* (73)]. In contrast, the mRNA levels of hormone-sensitive lipase, as well as lipoprotein lipase (*lpl*), decreased in the liver of Mozambique tilapia (*O. mossambicus*) in response to homologous hormone treatment (71). The latter study also observed a decrease in hepatic glycogen content and corresponding increase in plasma glucose (71), suggesting that leptin has hyperglycemic actions in teleosts and thus may represent a functional divergence from mammalian leptins. This corroborates an earlier study in rainbow trout (*O. mykiss*) in which central administration of leptin also increased plasma glucose while concurrently reducing the glycogen content of the liver (36). Similar effects were observed in lizards, with leptin decreasing hepatic glycogen content and increasing plasma glucose levels (74). Disparate results have been reported in goldfish, however, with human leptin increasing muscle and liver glycogen while depleting liver lipids and lowering plasma glucose, similar to what is observed in mammals (58). The different actions of leptin reported in teleosts could be a function of differences in life history strategies or from using mammalian vs. homologous leptins. Baltzegar et al. (71) reported similar glucoregulatory effects for both recombinant human leptin and tilapia LepA. However, distinct actions on regulation of hepatic *hsl* and *lpl* were observed between the two, with tilapia LepA reducing and human leptin having little effect on the lipases, suggesting that the use of homologous hormone may be essential for determining species-specific effects.

Further glucoregulatory roles for leptin have been demonstrated in the brain of rainbow trout and tilapia. Aguilar et al. (36) demonstrated increases in the glucose and glycogen contents of the trout (*O. mykiss*) hypothalamus and hindbrain in response to an intracerebroventricular injection of human leptin, which were paralleled by increases in *glut2* mRNA and glycogen synthase activity. Leptin also induced a significant increase in glucokinase activity in the brain (36), suggesting that one of the functions of leptin may be to stimulate glucose uptake and metabolism. In the pituitary rostral *pars distalis* of the tilapia (*O. mossambicus*), homologous leptin induced an increase in the activity of phosphofructokinase, a rate-limiting glycolytic enzyme, and this was correlated with an increase in lactate secretion or overall glycolytic output (75). Although typically believed to be a lipolytic agent, leptin has also been implicated in glucose metabolism in mammals, having been shown to stimulate glycolysis and gluconeogenesis and inhibit glycogenolysis [reviewed in Ref. (76)]. These data suggest that one of the basal functions of leptin may be to regulate glucose uptake and catabolism (e.g., glycolysis) in vertebrates; however, the source of glucose may vary as the hormone can elicit catabolic effects on either lipid or glycogen stores. One explanation for this could be the evolution of endothermy [see Ref. (77) for review of energetics between endothermy and ectothermy]. Mammals exhibit higher metabolic rates that, if fueled by fatty acids and/or glucose that has been synthesized *de novo*, would allow glycogen stores to be conserved in the event the animal is in need of a rapid source of energy. Hence, leptin may promote gluconeogenesis, but not glycogenolysis. Whether leptin alters gluconeogenic pathways in fish remains to be determined.

## Leptin Integration with the Classical Endocrine Stress Axis

### Endocrine Stress Response

It is apparent that leptin is a catabolic hormone in vertebrates that enhances energy mobilization and suppresses appetite, two processes often linked to stress responses. Hence, the hormone may be integral to the endocrine stress response. Stress impacts virtually all aspects of vertebrate physiology including immunity, reproduction, hydromineral balance, and energy homeostasis (78–80). The adrenergic (humoral and neuronal) and hypothalamic–pituitary–adrenal [interrenal in fish; HPA/hypothalamic-pituitary interrenal (HPI)] axes are central components of the vertebrate stress response and ultimately aid in restoration of homeostasis when disrupted. In all vertebrates, including teleost fishes, acute and chronic stress events are mediated through the sympathetic adrenergic and HPA/HPI axes, two primary components of the endocrine stress response. The two axes release catecholamines (epinephrine/norepinephrine) and glucocorticoids (cortisol/corticosterone), respectively, to allow for the mobilization of energy stores (79, 81, 82).

Upon the perception of a stressor, sympathetic nerve fibers release acetylcholine onto chromaffin cells within the adrenal medulla (mammals) or interrenal tissue (teleosts) to stimulate the secretion of catecholamines and allow for the rapid mobilization of energy stores from peripheral tissues (81, 83–85). Simultaneously, the hypothalamus releases corticotropin-releasing factor (CRF), which stimulates the release of adrenocorticotropic hormone (ACTH) from the pituitary. ACTH then triggers the production and release of glucocorticoids from the adrenal cortex (mammals) or interrenal cells of the head kidney (teleosts) (79, 80, 85). These glucocorticoids then elicit a myriad of metabolic effects such as inducing lipid and protein catabolism and stimulating gluconeogenesis to increase plasma glucose levels (79, 86). In a classic negative feedback pathway, the increase in circulating cortisol then inhibits further release of CRF and ACTH, attenuating the stress response.

### Catecholamines and Leptin

**Figure 3:**
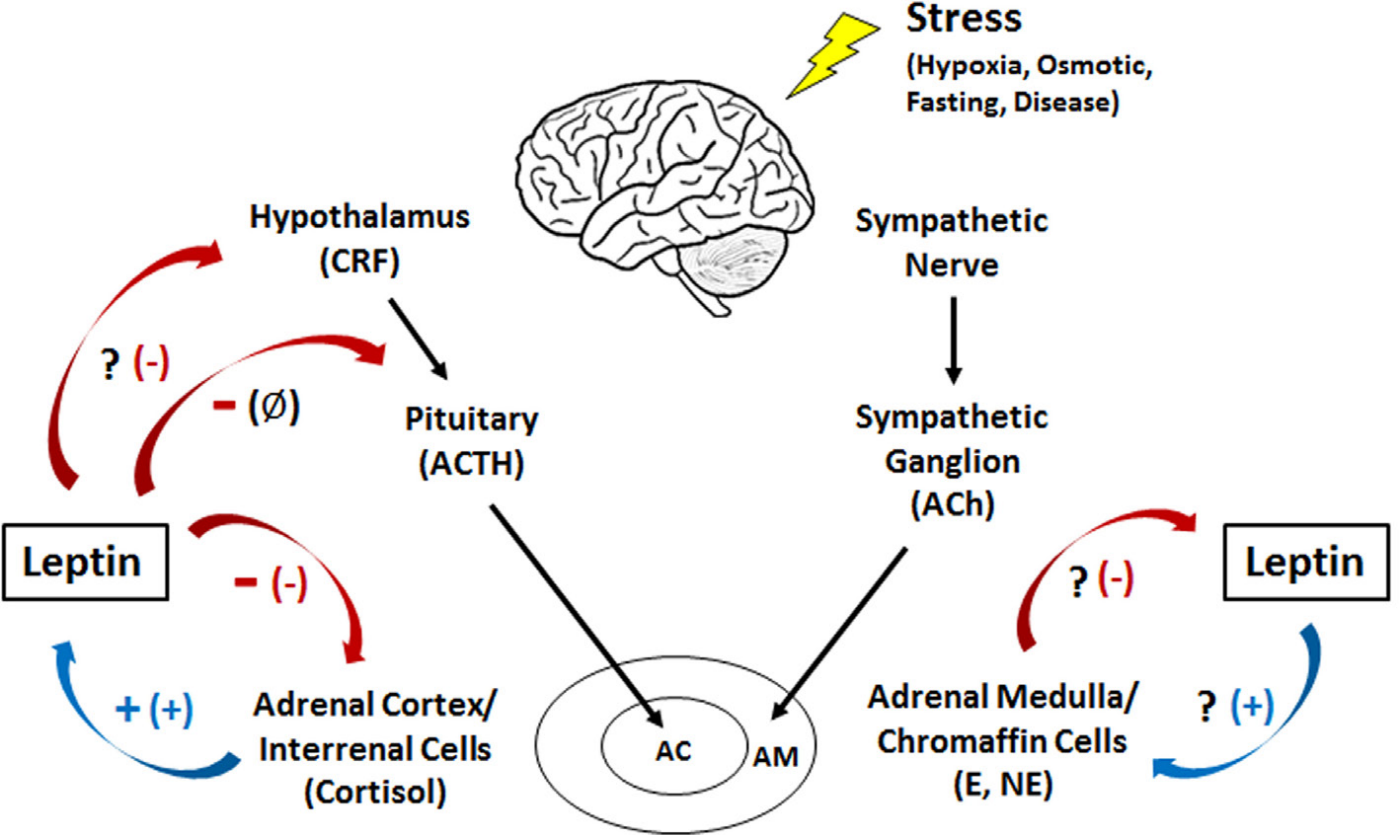
**Interactions between leptin and the humoral adrenergic and hypothalamic–pituitary–adrenal/interrenal axes in teleosts and mammals**. +, stimulation; -, inhibition; Ø, no effect; ?, an unknown relationship. The mammalian response is represented by the symbol in parentheses. CRF, corticotropin-releasing factor; ACTH, adrenocorticotropic hormone; Ach, acetylcholine; E, epinephrine; NE, norepinephrine; AC, adrenal cortex; AM, adrenal medulla.

Epinephrine is thought to be the primary hormone of the humoral adrenergic system in most fishes (80, 81). As part of the “fight or flight” response, catecholamines exert numerous actions that include rapid mobilization of glucose and free fatty acids through enhanced glycogenolysis and lipolysis, respectively, as well as regulation of respiration and blood flow (79, 81, 87). Leptin is also critical for regulating energy expenditure in vertebrates and responds to various stressors (see Leptin Responses to Stress in Vertebrates), yet little is known about how the hormone interacts with components of the endocrine stress axis, particularly in non-mammalian vertebrates (27). To date, the majority of studies examining the relationship between leptin and the stress axis have been performed in mammals (27, 51). However, studies in lizards [*Podarcis sicula* (74)] and teleosts have indicated that leptin may act as a key metabolic regulator during stress in all vertebrates through mobilization of energy stores (Figure 3).

Leptin has been shown to stimulate the release of catecholamines in both porcine (88) and bovine (89) adrenal medullary cells. In addition, leptin increased mRNA levels of tyrosine hydroxylase, the rate-limiting enzyme in catecholamine production (88). This suggests a synergistic relationship between leptin and catecholamines wherein leptin mobilizes energy from lipids while simultaneously stimulating the release of catecholamines to mobilize glucose during periods of stress (90). Interestingly, other studies utilizing human chromaffin cells have shown no significant change in catecholamine release with leptin treatment (91). The contradictory responses observed between human and other mammalian models could possibly be due to differences in methodology (isolated cells vs. whole adrenal tissue), the leptin concentrations used, or simply species differences (88). The regulation of catecholamines by leptin in fishes has not been well characterized. In goldfish (*C. auratus*), chronic leptin treatment resulted in no significant changes in hypothalamic catecholamines (58); however the effects of leptin on circulating catecholamines are yet to be examined.

While leptin exerts a stimulatory effect on catecholamine release in mammals, epinephrine has been shown to directly inhibit leptin secretion (92–95). In addition, increases in intracellular cAMP in medullary cells, one of the second messengers involved in adrenergic signaling, downregulate leptin mRNA (96). Leptin increases intracellular cAMP in addition to stimulating catecholamine release (88), both of which could act in a negative feedback loop to inhibit further leptin release. One theory behind this inhibition is that it is not advantageous for catecholamines to stimulate leptin during acute stress as obtaining energy from lipolysis is too slow for a “fight or flight” response; however, it may play a role in mediating the response to chronic stress (27). The regulation of leptin by catecholamines in fishes and other ectotherms is still unclear. However, both leptin and epinephrine exhibit glycogenolytic and/or lipolytic actions and have been shown to increase during times of stress in fishes (71, 79).

### Glucocorticoids and Leptin

The human *ob* promoter region possesses glucocorticoid response elements, suggesting that cortisol may elicit some of their actions by inducing leptin transcription (97, 98). Indeed, glucocorticoids elicited a stimulatory effect on leptin synthesis and secretion in rats (99), humans (100), and cultured human adipocytes (101) (Figure 3). In addition, the synthetic glucocorticoid dexamethasone increased mRNA levels and stimulated leptin secretion in rat adipocytes (96, 102). Similar results have been observed in teleosts, with cortisol increasing hepatic leptin mRNA levels in rainbow trout (*O. mykiss*) both *in vivo* and *in vitro* (103). In addition, when trout hepatocytes were treated simultaneously with cortisol and RU486, a glucocorticoid receptor antagonist, the increase in leptin mRNA was attenuated (103). Whether a similar response occurs with leptin secretion remains unknown. It has been speculated that since cortisol release is slower than that of catecholamines, the prolonged stressors that elicit cortisol actions would also benefit from the catabolic effects of leptin on lipids and/or carbohydrates reported in fishes, particularly in the liver where leptin is produced and may act locally (26, 27, 39, 71, 73, 104).

Leptin in turn has an overall inhibitory effect on the HPA axis in mammals (98), inhibiting CRF release from the hypothalamus in mice (105) and suppressing cortisol secretion from adrenal cells (106–108) (Figure 3). In contrast, leptin has no effect on ACTH secretion from the pituitary, suggesting that it regulates glucocorticoid release indirectly *via* the hypothalamus and directly by acting on the adrenal gland (105). When human adrenocortical cells are incubated with leptin, a dose-dependent decrease in ACTH-stimulated cortisol secretion is observed (91), while in leptin knockout mice (*ob/ob*), circulating levels of glucocorticoids are 85% higher than basal. Injecting these knockouts with leptin, however, reduced the level of glucocorticoids by 40% (109, 110). These data could potentially suggest a synergism between leptin and cortisol wherein cortisol stimulates the secretion of leptin that, in turn, mobilizes energy stores necessary for coping with a stressor. It has also been suggested that the anorexigenic effects of leptin could counteract the weight gain effects of cortisol in mammals (111). Similar results have been observed in teleosts, suggesting that interactions between leptin and glucocorticoids may be conserved in vertebrates. In the common carp (*C. carpio*), leptin inhibited ACTH-stimulated cortisol secretion *in vivo* and caused a dose-dependent decrease in CRF-induced ACTH secretion from the pituitary *in vitro* (6, 112). No changes in circulating cortisol were observed in leptin-injected goldfish [*C. auratus* (59)]; however, it is possible that leptin only inhibits glucocorticoid production when the HPI axis has been activated and circulating cortisol levels are elevated. In general, we do know that teleost pituitary glands are responsive to leptin (6, 26, 40, 42), and as such, it has been postulated that leptin may regulate the stress axis at the level of the pituitary (6, 113).

Currently, there are no other studies in fishes examining the relationship between leptin and the hormones of the stress axis, specifically interactions with catecholamines and glucocorticoids. There is a need to address these gaps as understanding these interactions will help to elucidate leptin’s basal function as a putative regulator of the endocrine stress response in these organisms and how these actions may differ from that of the classically described adipostat in mammals.

## Leptin Responses to Stress in Vertebrates

### Fasting

Catabolic stress associated with fasting typically leads to downregulation of leptin expression in mammals (114). The preponderance of evidence in teleosts, however, points to fasting-induced increases in leptin synthesis and secretion (23, 42, 58, 72, 115, 116); albeit evidence in two species, the hybrid striped bass (*M. chrysops x M. saxatilis*) and red-bellied piranha (*Pygocentrus nattereri*) show that production of the hormone may decline with fasting (10, 117). The general increase in leptin during fasting found in most teleosts presents a functional paradox between the role of leptin as an anorexigenic endocrine signal and the drive to increase food intake during fasting. Leptin could aid to limit feeding to avoid the metabolic costs associated with foraging and digestion (118) during periods of low food availability, or perhaps other orexigenic factors such as ghrelin, whose levels are known to increase dramatically with fasting (119), outweigh the anorexigenic properties of leptin in driving food intake when energy status is low. Regardless, the increase in leptin with fasting is likely critical for promoting the catabolism of energy stores to fuel essential cellular processes. The variability of responses in fishes compared to mammals may be attributed to distinct regulation of energy stores, perhaps suggesting that signaling during altered metabolic states may not be reliant solely on leptin, but an integration of lipostatic, glucostatic, and other metabolic and endocrine signals. Further, as a consequence of genome duplication events in teleosts [reviewed in Ref. (120, 121)], some species possess multiple leptin paralogs that may exhibit different functional properties.

### Hyperosmotic Stress

Euryhaline fishes can withstand wide fluctuations in environmental salinity. Through active excretion of ions, they can overcome large increases in plasma osmolality (>150 mOsm) during acute seawater challenge (71). The process of seawater acclimation consumes 20–68% of their total metabolic energy demand (122, 123). Elevated leptin stimulates Na^+^ retention and induces hypertension in rats and may be associated with hypertension induced kidney disease in humans (124). Few studies have investigated the role of leptin in osmoregulation in teleost fishes, despite its regulatory interactions with GH, IGFs, and PRL, hormones known to control salt and water balance (26, 40–42). In the Mozambique tilapia (*O. mossambicus*), acute seawater transfer induced significant increases in hepatic *lepa* and *lepr* mRNA levels (71). The authors propose that leptin may work with cortisol to mobilize energy stores by inducing hepatic glycogenolysis and gluconeogenesis, respectively, thereby allowing the organism to fuel the increased energy demands associated with hyperosmotic stress. The hormone had no direct effect on expression of the gill Na^+^K^+^-ATPase pump, so it remains unclear whether the hormone is ionoregulatory in teleosts. Additional studies suggest that leptin may stimulate the release of PRL, an important freshwater osmoregulatory or Na^+^-retaining hormone in teleosts (26, 40). Collectively, the results suggest that leptin may act to mobilize energy for seawater adaptation and promote GH sensitivity and IGF production to enhance seawater acclimation (41, 42, 71). It may also promote synthesis and secretion of PRL for freshwater adaptation (40).

### Hypoxia

Oxygen is a necessary component of energy production in all vertebrates, and thus hypoxia represents a severe and potentially lethal stress. As leptin functions at the intersection of the endocrine stress response and metabolism, it is reasonable to postulate that it is involved in the vertebrate response to hypoxia. Indeed, an increase in the transcription of leptin in humans, observed in response to hypoxia and hypoxia-inducible factor 1 (HIF-1), transactivates the human leptin gene promoter (125, 126). In addition, leptin mRNA levels increase in response to hypoxia in a variety of mammalian cell lines (127–129). Interestingly, Meissner et al. (130) reported that short-term hypoxia in rats had no effect on plasma leptin levels or expression in adipose tissue; however, leptin expression was increased in the liver, kidney, and lungs suggesting a unique metabolic role for leptin under hypoxic stress. Leptin has further been shown to attenuate apoptosis under hypoxic conditions and appears to be necessary for behavioral recovery following acute hypoxia (131, 132). Taken together, the data from mammals point to a crucial role for leptin as a multifaceted mediator of energy homeostasis during hypoxia.

The first report of leptin regulation by hypoxia in fishes came from Chu et al. (133). The authors showed that *lepa* expression increased after 4 and 10 days of hypoxic exposure in zebrafish (*D. rerio*) and implicated HIF-1α as a key mediator of this response. In common carp (*C. carpio*), the expression of *lep-a1, lep-a2*, and *lepr* in the liver increases in proportion with the length of hypoxic exposure (113). This study also showed that exposure to hypoxia upregulated expression of *lepr* mRNA in the pituitary, suggesting potential integration with the HPI axis (113). In addition, transcriptome data for the tilapia (*O. mossambicus*) shows upregulation of genes responsive to hypoxia in the pituitary following leptin treatment [e.g., chaperone-containing TCP1, chromodomain helicase-binding domain, heat shock protein 90b1, Gene Ontology 0070482/001666 (75)]. Crucian carp (*C. carassius*) expresses multiple isoforms of the LepR in the gill, and the mRNA levels increased in response to hypoxia *in vivo* (134). While there are still significant gaps in knowledge with regards to how leptin is acting to augment organism energetics during hypoxia in fishes, it appears that leptin is indeed regulated by hypoxia in much the same way as mammals, increasing in response to the decreased availability of oxygen for ATP production. The emerging role of leptin in stimulating glycolysis among different vertebrates may fit well with its upregulation during hypoxia or normoxia (Warburg effect).

### Immune Function and Disease

Immunity is intimately linked to an organism’s metabolism and energy status, and as such, allocating energy to the immune system in states of both health and disease is critical to the overall fitness and survival of an organism (135). Fasting and nutritional deprivation are associated with an increased disease susceptibility, as well as immune system suppression and dysfunction in vertebrates (136–138). Due to its role as a vital neuroendocrine mediator of metabolic state, leptin has been investigated as a regulator of the energetics associated with the innate and adaptive immune responses. In mammals, increases in serum leptin levels occur with inflammation, a response that appears to be modulated by glucocorticoids (139). Further, leptin has been shown to reverse starvation-induced immunosuppression by stimulating the proliferation of pro-inflammatory cytokine-secreting T cells (140). Despite having been extensively studied in mammals, few studies have explored the interplay of leptin and immunity in teleost fishes or other non-mammalian vertebrates.

The correct allocation of energy to the innate immune system, the first line of organism defense and the most important responder in the acute phase of an infection, is critical to host survival. Leptin signaling has been shown to be necessary for innate immunity in mammals (135, 141), increasing chemotaxis and oxidative function and delaying apoptosis in immune cells (142–146). Leptin increases activation and proliferation and induces production of pro-inflammatory cytokines in phagocytes (147). Similar functions have been observed in the adaptive immune response, wherein leptin acts to stimulate B-lymphocytes by inducing cell cycle entry, preventing apoptosis and causing the secretion of pro-inflammatory cytokines (148, 149). In addition, it has been determined that leptin signaling is necessary for normal rates of glucose uptake and glycolysis in activated T-cells (150). These data suggest that, in mammals, leptin may drive immune activation by increasing the oxidative and overall glycolytic capacities of various immune cells.

Very little work has been done to directly connect leptin to the immune system in teleost fishes. Mariano et al. (151) showed that leptin drove ERK and STAT3 phosphorylation in both adherent and non-adherent trout leukocytes. Additional evidence for a role of leptin in regulating immune function in teleosts comes from MacDonald et al. (152) in which rainbow trout (*O. mykiss*) infected with a pathogenic hemoflagellate exhibited significantly higher mRNA and plasma levels of LepA. The authors determined that leptin was being secreted in response to the hypoxemia associated with the infection to reduce food intake (152). This would serve to prevent the organism from having to allocate energy toward digestive functions while in the diseased state. It is also possible that increases in leptin synthesis and secretion could lead to catabolism of energy stores necessary to meet the energetic demands of fighting the disease. Although limited, the data suggest an integration of leptin with immune function, and future studies should investigate the extent of leptin’s involvement in immunometabolic pathways in teleost fishes.

## Conclusion

In teleost fishes, there is much that remains to be elucidated about the role of leptin in energy homeostasis. Although there is evidence that leptin acts as a glucoregulatory agent in teleosts, there are also reports of leptin having lipolytic actions, particularly in the cyprinid fishes. In mammals, leptin has been implicated in regulating the metabolism of both glucose and lipids, suggesting some conservation of function between the two groups, perhaps sharing roles in promoting glycolysis. However, the increase in leptin levels during fasting presents a functional paradox against its role as an anorexigenic hormone. A further look into the function of leptins in regulating basal metabolism may shed light in this area. As multiple paralogs of leptin have been identified in teleosts, future studies should focus on whether the disparate actions are simply species-specific differences or the result of neofunctionalization between the various leptin paralogs. To date, the studies investigating the involvement of leptin in regulating immunity and the endocrine stress response suggest that such roles may be conserved within vertebrates. However, it is currently unclear by what means metabolic energy stores might be preferentially mobilized by leptin upon exposure to acute and chronic stressors, such as osmotic stress or hypoxia. Further, it remains to be determined how multiple endocrine signals (e.g., catecholamines, glucocorticoids) might integrate with leptin signaling to achieve the appropriate physiological response under such conditions. Studies in teleosts, or other ectotherms, may shed light on potential new functions of leptin that may be well conserved in the vertebrate lineage.

## Author Contributions

All individuals contributed to writing and reviewing the final version of the article.

## Conflict of Interest Statement

The authors declare that the research was conducted in the absence of any commercial or financial relationships that could be construed as a potential conflict of interest.
